# Nanostructured scaffold as a determinant of stem cell fate

**DOI:** 10.1186/s13287-016-0440-y

**Published:** 2016-12-30

**Authors:** Lekshmi Krishna, Kamesh Dhamodaran, Chaitra Jayadev, Kaushik Chatterjee, Rohit Shetty, S. S. Khora, Debashish Das

**Affiliations:** 1 Stem Cell Research Lab, GROW Lab, Narayana Nethralaya Foundation, Bangalore, Karnataka India; 2 School of Bioscience and Technology, VIT University, Vellore, Tamil Nadu India; 3 Vitreoretina Services, Narayana Nethralaya Eye Hospital, Bangalore, Karnataka India; 4 Department of Materials Engineering, Indian Institute of Science, Bangalore, Karnataka India; 5 Department of Cornea and Refractive Surgery, Narayana Nethralaya Eye Hospital, Bangalore, Karnataka India

**Keywords:** Biomaterial, Stem cell, Differentiation, Architecture, Scaffold

## Abstract

The functionality of stem cells is tightly regulated by cues from the niche, comprising both intrinsic and extrinsic cell signals. Besides chemical and growth factors, biophysical signals are important components of extrinsic signals that dictate the stem cell properties. The materials used in the fabrication of scaffolds provide the chemical cues whereas the shape of the scaffolds provides the biophysical cues. The effect of the chemical composition of the scaffolds on stem cell fate is well researched. Biophysical signals such as nanotopography, mechanical forces, stiffness of the matrix, and roughness of the biomaterial influence the fate of stem cells. However, not much is known about their role in signaling crosstalk, stem cell maintenance, and directed differentiation. Among the various techniques for scaffold design, nanotechnology has special significance. The role of nanoscale topography in scaffold design for the regulation of stem cell behavior has gained importance in regenerative medicine. Nanotechnology allows manipulation of highly advanced surfaces/scaffolds for optimal regulation of cellular behavior. Techniques such as electrospinning, soft lithography, microfluidics, carbon nanotubes, and nanostructured hydrogel are described in this review, along with their potential usage in regenerative medicine. We have also provided a brief insight into the potential signaling crosstalk that is triggered by nanomaterials that dictate a specific outcome of stem cells. This concise review compiles recent developments in nanoscale architecture and its importance in directing stem cell differentiation for prospective therapeutic applications.

## Background

The critical feature of stem cells is their ability to proliferate and differentiate using niche-dependent cues provided by signaling molecules, intercellular communication, and their neighboring extracellular matrix (ECM). Any of these components can be modulated to obtain specific lineage outcomes [1].

The insight in this review would provide reasonable approaches for researchers and clinicians to obtain a programmed cellular lineage by biomaterial structural modifications.

## Stem cells and biomaterials

A key area of research that has gained significant attention over the past several years is tissue engineering—an allied field of regenerative medicine. The science of biomaterials has evolved from a cell carrier tool to one that can direct cellular differentiation. Biomaterials can now be molded into three-dimensional (3D) scaffolds to promote cell proliferation and/or differentiation for regeneration [2]. Mechanical factors such as matrix stiffness, matrix nanotopography, microgeometry, and extracellular forces significantly influence stem cell activities. Based on the source of derivation, biomaterials can be grouped under natural and synthetic polymers. Some of the natural scaffolds used in tissue engineering include collagen, silk fibroin, alginate, chitosan, keratin, and decellularized tissues such as de-epithelialized human amniotic membrane [3]. Biodegradability and a biologically active nature are the major advantages of natural scaffolds over synthetic scaffolds. Cells cultured on natural scaffolds reveal a good cellular response with enhanced tissue growth and host tissue integration on transplantation. One of the major drawbacks of natural scaffolds is their inherent ability to become cross contaminated from the source.

Synthetic scaffolds represent the largest group of biodegradable polymers with consistent properties apart from a high surface to volume ratio, versatility in chemical composition, and biological properties that show good malleability and processability [4, 5]. Polymers of diverse properties have been used for fabrication of scaffolds to be used for different applications. One of the major drawback of the synthetic scaffolds is the local inflammation initiated by the release of acids as their degradation byproduct [5].

## Influence of the biophysical microenvironment on stem cell response

**Fig. 1:**
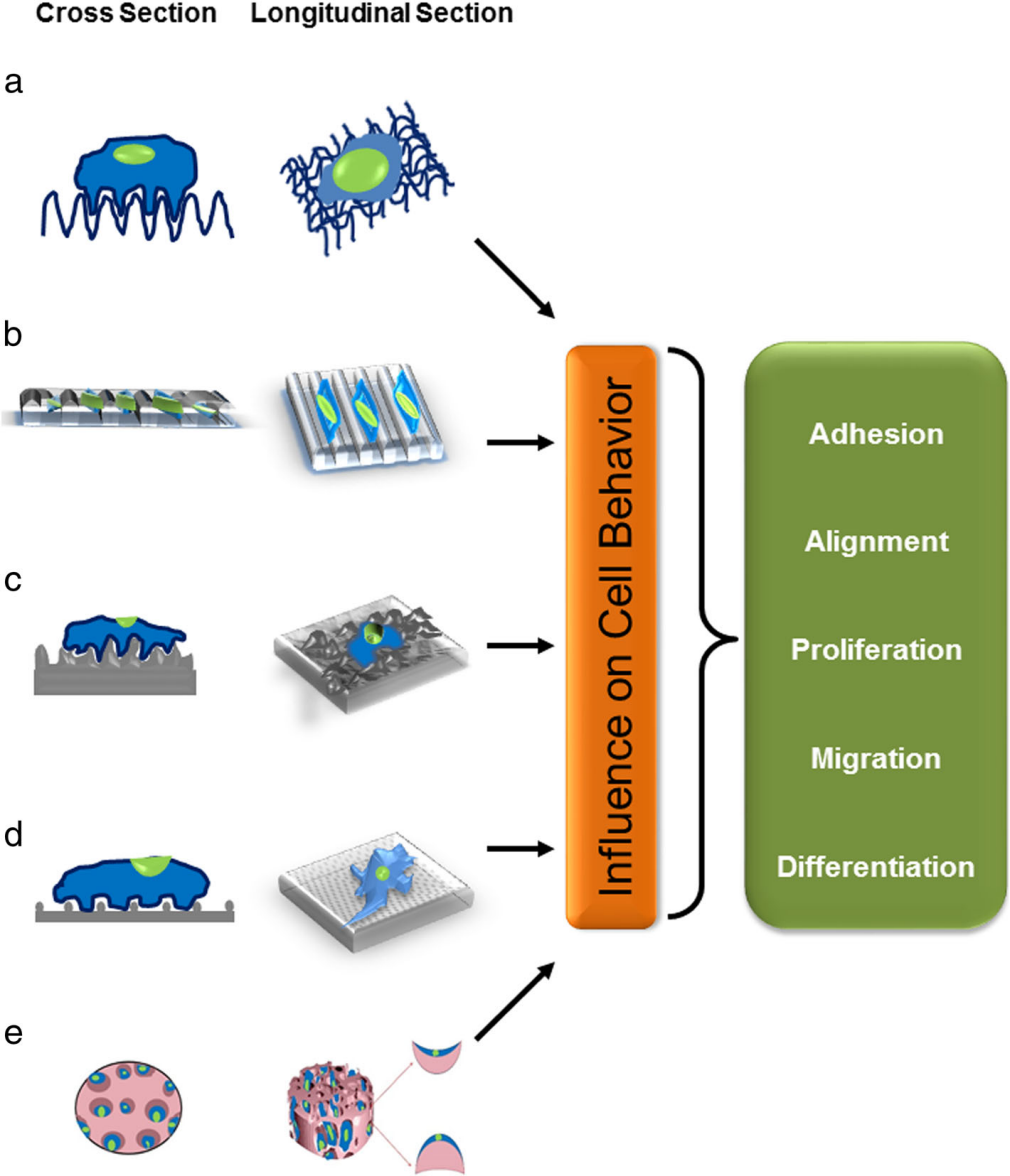
Cellular response to the biophysical microenvironment. Biomaterials with (**a**) fibrous architecture, (**b**) nano grooves/ridges, (**c**) surface roughness and varying nanotopographical features, (**d**) nanodotted surface, and (**e**) concave and convex curvatures inside a porous scaffold. These microenvironmental mechanical cues have the ability to influence cell adhesion, alignment, proliferation, differentiation, and migration

A cell responds to its environmental cues through the cellular mechanotransduction pathway. The soluble and insoluble cues regulate/modulate various genes and their downstream effectors. The physiological outcome of a cell growing on a scaffold is defined by three factors—biological, biochemical, and biomaterial. [6]. Different techniques with different architectures are used for synthesizing scaffolds for a specific biological or clinical application. (Figure 1). In the following section we have listed a few methods that impart architectural uniqueness to scaffold design and their limitations with respect to stem cell applications.

### Nanoscale platforms

One of the major challenges in biomaterial science is to generate a substrate topography that mimics the in vivo microenvironment composed of pores, ridges, and channels that provide physical cues to cells at a nanoscale level. Scaffolds are generated by the techniques described below.

#### Electrospinning

Electrospinning is one of the most widely used fabrication techniques. Nanofibers of sub-microscale diameter are generated by ejecting electrically charged polymer solutions through a needle on to a grounded collector surface [7]. Usage-dependent customized nanofibers of different architectures and shapes can be fabricated using patterned collectors on electrospinning. Since the fiber diameter is lesser than the cell surface area, it is a perfect platform for the cells to organize and spread around the nanofibers with numerous focal adhesion points [6]. Pores of variable sizes can be introduced during the generation of electrospun scaffolds by incorporating porogens or sacrificial fibers that get dissolved after fabrication [8, 9]. A wide range of polymers of both natural and synthetic materials are employed for manufacturing scaffolds [10]. Though the biological materials promise better clinical applications it is difficult to maintain the integral chemical features of the material during the electrospinning process. For example, collagen loses its physicochemical properties when fluoroalcohol is used as a solvent to generate nanofibers [11]. It is much more challenging to precisely control the dimension and morphology of silk fibroin as 3D electrospun scaffolds for specific biomedical and dental applications [12]. Some drawbacks of this method are the possibility of cells squeezing into smaller pores thereby limiting their growth potential and the lack of precise control of fiber alignment. Modifications to the electric charges and the introduction of high-speed rotation mandrels have overcome this limitation [13]. Cells seeded on a nanofiber structure tend to maintain the phenotypic shape and a guided growth according to nanofiber orientation. Yin et al. differentiated human tendon stem/progenitor cells to tendons using electrospun poly-l-lactic acid (PLLA) nanofibers [14]. The cellular morphology was defined by the nano-architecture; cells were more stellate-patterned on random nanofibers and elongated and spindle-shaped fibroblastic phenotype with the aligned nanofibers [14]. Independent of the differentiation factors, the scaffold architecture is capable of directing lineage specification. For example, human bone marrow stromal cells (hBMSCs) cultured on nanofibrous poly-ε-caprolactone (PCL) scaffolds adopted an elongated, highly branched, osteogenic morphology [15]. Morelli et al. have reported that polylactic acid (PLA) and composite PLA-nanohydroxyapatite electrospun scaffolds were equally efficient in differentiating human mesenchymal stem cells (MSCs) to osteogenic and osteoclastogenic differentiation [16]. Kai et al. in a recent study demonstrated that electrospun composite PCL-gelatin scaffolds encapsulated with vascular endothelial growth factor promoted differentiation of MSCs for myocardial regeneration [17]. Furthermore, Mohtaram et al. found that electrospun PCL scaffolds with a smaller diameter loop mesh induced higher neuronal differentiation compared to thicker loops from neural progenitors [18]. Electrospun nanofibrous scaffolds of polylactic-co-glycolic acid (PLGA) and gelatin with embedded epidermal growth factors have been used for tissue engineered skin scaffolds [19]. Recently, Ortega et al. used a combination approach of electrospinning and microstereolithography to generate corneal membranes that mimicked the limbal region of the eye that harbor ocular stem cells [20]. Embedding guidance cues with gradient concentration on electrospun fibers can provide a polarized effect on the cultured cells and tissues. Similarly, techniques such as air brushing are now being used to eliminate the use of organic solvents for the preparation of polymer solutions. One of the salient feature of electrospinning is controlling the fiber alignment, which has been achieved for proper control of cellular functions through control of cellular morphology and alignment [21, 22]. The release of encapsulated drugs and biomolecules is being tailored through the use of core-shell fibers [23].

Though the technique is widely popular, the process of electrospinning depends on the polymer solution properties such as viscosity, surface tension, conductivity, and dielectric constant [24]. The viscosity of the solution maintains the ejecting fiber without breakages [25]. Voltage applied, flow rate of solution, type of collector, needle diameter, and the distance between the needle and the collector are factors that determine the pattern of the fibers [26]. Environmental factors such as temperature, humidity, and pressure can have some minor effects on the patterning of fibers by electrospinning [27]. The number of factors affecting the scaffold outcome generated by electrospinning is numerous, making it difficult to form a standard operating protocol for repeatability of scaffold architecture. The infiltration of cells within electrospun fibers is rather limited. The fibers are typically unable to serve as scaffolds for load-bearing tissues [28].

#### Soft lithography

Soft lithography fabrication uses elastomeric stamps, molds, and conformable photomasks ranging from micrometer to nanometer scale for scaffold generation. The synthesizer, based on the application, can customize the spatial distribution of polymer molecules placed on the substrate to aid specific outcomes such as nanodots, nanoridges, and grooves in the range from 30 nm to several microns [29, 30]. This spatial distribution of the polymer molecules also aids in spreading and shaping individual as well as groups of cells [31–33]. Embryonic stem cells are cultured in embryoid bodies and further differentiated using conditioned culture methods for lineage specificity [34, 35]. In an attempt to identify a stem cell delivery system, murine muscle satellite cells were cultured on 3D polyglycolic acid (PGA) scaffolds fabricated from a combination of soft lithography and thermal membrane lamination. Cells delivered by scaffold show higher integration to the damaged tibialis anterior muscles in comparison to cells injected intramuscularly [36]. Grooved patterns of micro- or nanoscale structures promote cell alignment and differentiation, especially with human embryonic stem cells, into neuronal lineage without the need for any supplements [37]. Hollow spheres are fabricated by injecting liquid drops into noncured polydimethylsiloxane (PDMS) mixtures. Furthermore, such drops provide a cell culture environment for growing embryoid bodies [38]. Micropatterned PDMS scaffolds generated by soft lithography have been used for mimicking musculoskeletal junctions connecting aligned myotubes with acetylcholine receptors [39]. However, the major limitation with this technique is that it provides a limited and narrow range of ECM signals for the cells to perceive, which can be highly inconsistent in comparison to the vast in vivo microenvironmental cues. This is further substantiated by the in vitro study where PLGA substrates of different groove depth promoted human tenocyte alignment with simultaneous upregulation in the expression of chondrogenic and osteogenic genes. On the contrary, in a rat patellar tendon model, neither of the grooved topographies induced ECM orientation parallel to the substrate. This indicates that cell phenotype maintenance is well established by two-dimensional (2D) imprinting technologies only in an in vitro condition. In an in vivo scenario, the neotissue formation and organization is established by multiple factors [40]. Another study deciphered the 2D imprinting technique exclusively to assess cell function in vitro for phenotype maintenance of human primary osteoblast phenotype on substrates of different grooves. Furthermore, in the in vivo sheep model, none of these topographies promoted osteogenesis [41].

Soft lithography is limited by distortion in the fabrication of single-layer structures [42]. The defects formed, which arise from dust particles, poor adhesion to the substrate and poor release from the stamp, must be controlled. Another drawback is the formation of a thin film of polymer under the nanometer-sized features. This layer is removed through ion etching but leads to damage of small nano-features generated on the fabricated scaffold. Integration of large and small features in phase-shift lithography is extremely difficult [43].

#### Microfluidics

Microfluidic devices make an excellent platform to study cells under various microenvironmental conditions such as stress capillary flow, chemical gradients, and the effects of single/low cell numbers on the temporal and spatial resolution. In microfluidics, the capillary flow maintains a constant soluble microenvironment and has a large surface area to volume ratio similar to biological systems [44]. This has been used extensively to study cell biological aspects such as cellular adhesion forces, the cytoskeleton, and for in vitro culture techniques. Microfluidics are used for high-throughput screening because of the capacity to culture a limited number of cells in a controlled manner. Such a system can thereby standardize culture conditions for differentiation without altering the cell number. Major limitations of microfluidics in long-term stem cell cultures are because of liquid evaporation, protein adsorption, leaching of non-reactive compounds, and hydrophobic recovery. Despite the aforementioned limitations, microfluidics provide a potential for simultaneous multi-parametric analysis with respect to the differentiation paradigm [45]. Mouse embryonic stem cell (mESC) differentiation studies using microfluidic systems have elucidated the decisive roles of fibroblast growth factor (FGF)4 and notch signaling during neuroectodermal lineage [46]. Three-dimensional microfluidics mimic the in vivo situation more closely, hence this microenvironment would be ideal for studying organogenesis and differentiation.

Much like soft lithography, the field of microfluidics employs strategies wherein the cells are grown on a substrate in 2D format and subjected to fluid flow. Designing microfluidic systems for 3D scaffolds remains a challenge and is just starting to be investigated. This fluidic strategy has to be utilized much more for clinical application [47].

### Nanoparticles in nanotechnology

Nanoparticles have contributed immensely in altering the physicochemical properties of the scaffold because of their variable size and shapes. Most of the properties attributed to nanoparticles are driven by their high surface to volume ratio, improved solubility, electrical and heat conductivity, and improved catalytic activity on the surface [48]. These nanoparticles can be used for altering the scaffold architecture either by decorating the scaffold surface to impart surface features and varying surface chemistry as well as being incorporated in the matrix during scaffold synthesis to vary mechanical properties, electrical conductivity, and so forth. The most widely used nanoparticles in this field can be classified into five groups based on their nature: carbon based, inorganic base, metal based, nanostructured hydrogels and quantum dots based [49, 50].

#### Carbon nanotubes

Carbon nanotubes (CNTs) derived from graphene sheets are prepared with precise control of orientation, alignment, nanotube length, diameter, purity, and density. They are constructed as single-walled (SWNTs) or multi-walled carbon nanotubes (MWNTs). CNTs have tunable chemical and mechanical properties, like conductivity, biocompatibility, and nanoscale dimensions, that serve as topographical cues and to generate electrophysiological properties [51, 52]. Composites with polycarbonate membrane and collagen sponges promote the osteogenic potential of stem cells. Interactions with fibroblasts were noted to be enhanced in polyurethane composite scaffolds. Better adherence and enhanced proliferation could be observed in endothelial cells cultured on composite polyurethane scaffolds. Polyacrylic acid composites aided in neuronal differentiation from embryonic stem cells [52–56]. The major drawback of CNTs is the presence of impurities of carbonaceous particles such as nanocrystalline graphite, amorphous carbon, fullerenes, and different metals (typically Fe, Co, Mo or Ni) used as catalysts during the synthesis phase, and also concerns with toxicity as they are resistant to degradation in vivo [51, 57]. More recently, other forms of carbonaceous nanoparticles such as graphene and nanodiamonds are also being investigated [58, 59].

#### Metal and metal oxide nanoparticles

Metal oxide nanoparticles provide structural variabilities by exhibiting conductor or insulator characters. Oxide nanoparticles display unique chemical and physical properties with differential charge on the center and corner of the nanoparticle [60]. They have mostly been used in tracking stem cells post-transplantation [61–63]. MSCs incubated with magnetized iron oxide nanoparticles promoted calcium nodule formation in the presence of osteogenic culture medium [64]. Superparamagnetic iron oxide nanoparticles quench H_2_O_2_ and thereby promoted growth of MSCs [65]. Delcroix et al. showed that rat MSCs, when loaded with superparamagnetic iron oxide nanoparticles coated with 1-hydroxyethylidene-1.1-bisphosphonic acid and injected, showed migratory behavior only on creating a lesion [66]. Copper oxide nanoparticles did not show any effect on the differentiation potential of rat MSCs to osteogenic and chondrogenic lineage. Enhanced genotoxicity could be observed in the MSCs with increasing dosage of copper oxide nanoparticles [67].

#### Inorganic based

These are ceramic-based nanoparticles synthesized by a combination of a metal and a non-metal component. These are formed under higher temperature and pressure [68, 69]. These materials have high mechanical strength and low biodegradability. Hydroxyapatite and tricalcium phosphate nanoparticles have been shown to promote bone formation [70]. Silica nanoparticles enhance actin polymerization and promoted osteogenesis from MSCs [71]. Furthermore, these nanoparticles coated on scaffolds promote cellular growth of adipose-derived stem cells in culture through Erk kinase activation [72]. Fibrin-poly(lactide-caprolactone) nanoparticle-based scaffolds enhance the human adipose-derived stem cell seeding efficacy and promote cell growth and chondrogenic differentiation [73]. Embryonic stem cells cultured on polystyrene nanoparticles differ in their morphology based on their culture density. At lower density, the embryonic stem cells transform to embryoid bodies, whereas at higher density they became fibroblastic when cultured on polystyrene nanoparticles [74].

#### Quantum dots based

These are nano-sized semiconductors that can emit light in different colors. These comprise atoms for releasing electrons and cadmium as one of the chemicals. Most of their usage is limited to tracking stem cells undergoing differentiation and migration [75, 76]. These are photostable and have longer longevity. So far there have been no reports on the effect of quantum dots in altering stem cell proliferation or differentiation [77].

A major concern with the use of nanoparticles is their toxicity and environmental effects. The environmental effect during production of nanoparticles itself is a global concern [78]. Moreover, when used in scaffolds, the long-term effect in vivo is not well understood.

#### Nanostructured hydrogels

Hydrogels are 3D polymeric materials of a hydrophilic nature capable of holding large amounts of water. Co-polymerization/crosslinking free-radical polymerizations are commonly used to produce hydrogels by causing hydrophilic monomers to react with multifunctional crosslinkers to form a network. Hydrogels can be further classified into nanogels and micellar gels [79]. Nanogels are hydrophobic in nature and hence can be used to deliver products to cells. Nanostructured hydrogels are self-assembled injectable carriers of cells and proteins [80]. Chemically or physically crosslinked nanostructure scaffolds are fabricated by photo-irradiation of vinyl monomer conjugated to polyethlyne glycol, pluronic copolymers, and hyaluronic acid [80]. The degree of crosslinking determines the mechanical strength, durability, and swelling properties on the nanostructured hydrogels [81]. Most of the nanostructured hydrogels are primarily used for carrying genes and proteins to be delivered [82]. The environmental conditions are pivotal for crosslinking the monomers in a temperature- or pH-responsive crosslinking strategy [83]. This ability of the nanostructured hydrogels to transform from sol to gel form makes them the smart hydrogels. Mesenchymal stem cells cultured on nanostructured PEG-based hydrogels with nano-sized micelles showed an elevated gene expression of mesenchymal stem cell marker compared to those cultured without micelles [82]. Neural stem cells of human origin showed enhanced adhesion and proliferation when cultured on self-assembling peptide-based nanostructured hydrogels [84]. Nanostructured hydroxyapatite along with demineralized bone matrix were used for generating nanostructured hydrogels for growing mesenchymal stem cells. These cells showed increased osteocalcin production with alkaline phosphatase indicating higher osteogenic specific differentiation [85]. Nanostructured hydrogel with porous baghdadite shows sustained release of dexamethasone disodium phosphate, promoting osteogenic regeneration [86]. The in situ forming smart hydrogel can be functionalized by bioactive molecules to enhance growth and other functionality of stem cells. The sol-gel transition of the nanostructured hydrogels serve as carriers for drug and protein delivery in supporting regenerative medicine.

### The spatial shape and alignment in stem cell function

All techniques used for generating scaffolds provide a geometrical control of the morphology of cells. The effects of geometrical forces on cells are explained using theoretical models of network and continuum mechanical models. While a continuum mechanical model describes the distribution of adherent cells, a network model is based on the contractile cytoskeleton deciphering the relationship between the force distribution and shape of adherent cells.

**Fig. 2:**
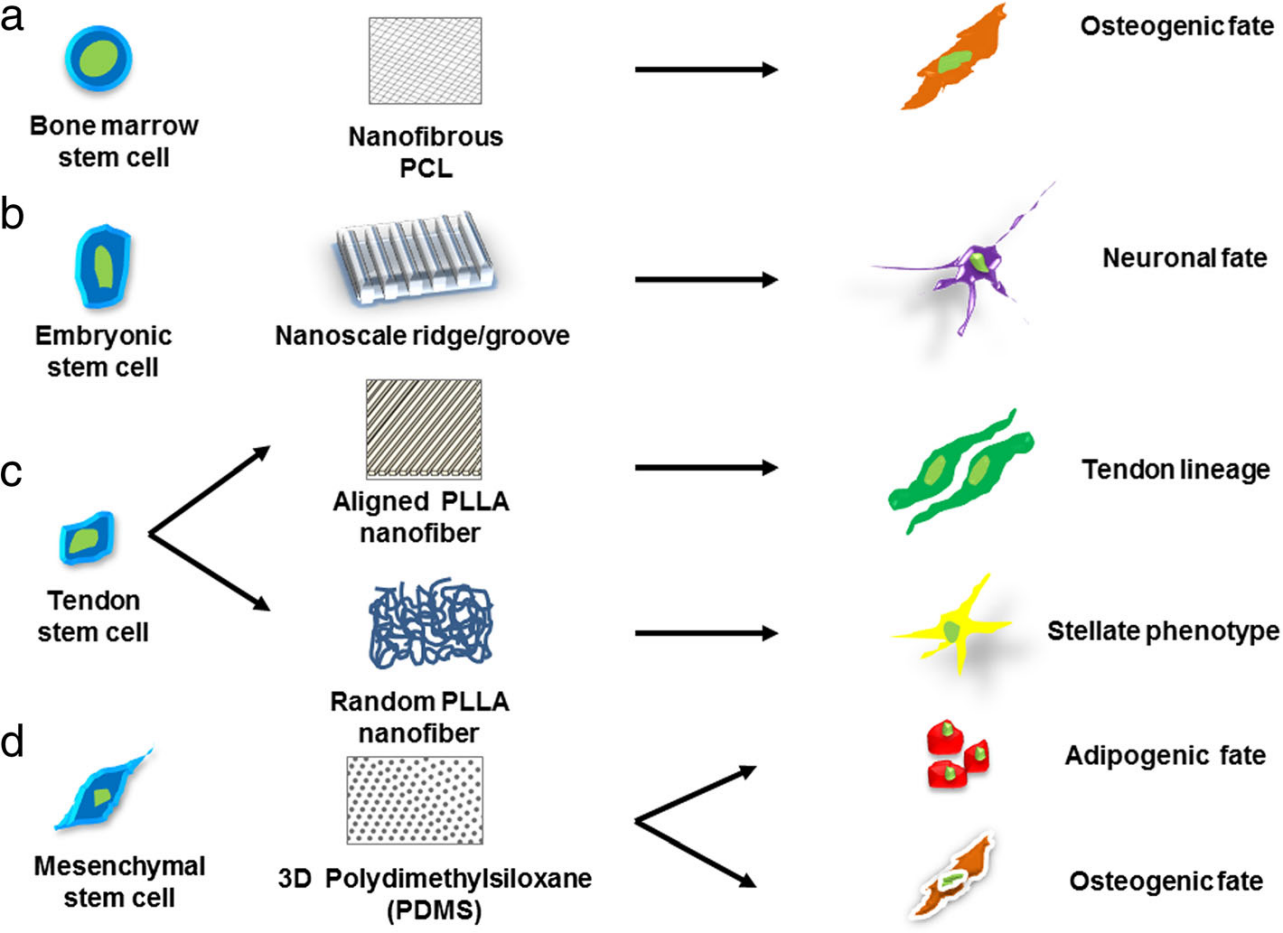
Schematic representation defining the importance of various scaffold architectures in determining the specific lineage of stem cells. Stem cells cultured on various nanostructured scaffolds yeild different differentiated cell types, such as **a** bone marrow stem cells grown on nanofibrous PCL scaffold promotes osteogenic fate, **b** embryonic stem cell cultured on annosclae ridge or groove promote neuronal fate, **c** tendon stem cells culutred on aligned and random PLLA directed tendon and stellate lineage, respectively, **d** mesenchymal stem cells on PDMS promote osteogenic as well as adipogenic fate.

Studies have revealed modulation of cellular functions such as proliferation and differentiation based on cell-specific locations in tissues with certain geometry. Stem cells in a 3D matrix respond to 3D architectural features at different scales from nanometers to micrometers and further to millimeters with differing functions in apoptosis, proliferation, and differentiation. The dominating effect of matrix geometrical force on cell fate incitement is pivotal in tissue-specific regeneration (Fig. 2).

Since cell shape and function are tightly linked together, scaffolds that modulate cell shape can dictate cell functions, for example the long body of neurons for effective delivery of signals, and the spherical shape of adipocytes for lipid storage. Human MSCs grown on microcontact-printed PDMS show osteogenic characteristics at the edges of the matrix and adipogenic nature towards the inner region of the scaffold [87].

Microenvironmental cues including mechanical forces are important for the formation of “stem cell niches”. Indeed, mechanical forces appear to either promote or block differentiation signals induced by growth factors and cytokines and they supersede the influence of soluble factors. To investigate the effects of mechanical forces on MSC differentiation, Kurpinski et al. used a micropatterned strip to align the cells along the direction of the uniaxial strain. They found an increased expression of calponin 1 (a smooth muscle marker) and a decrease in the expression of cartilage matrix marker. However, when the cells were aligned perpendicularly to the direction of the strain, the changes in gene expression were diminished [88]. This experiment suggests that mechanical strain has an important role in gene expression and fate of stem cells.

**Fig. 3:**
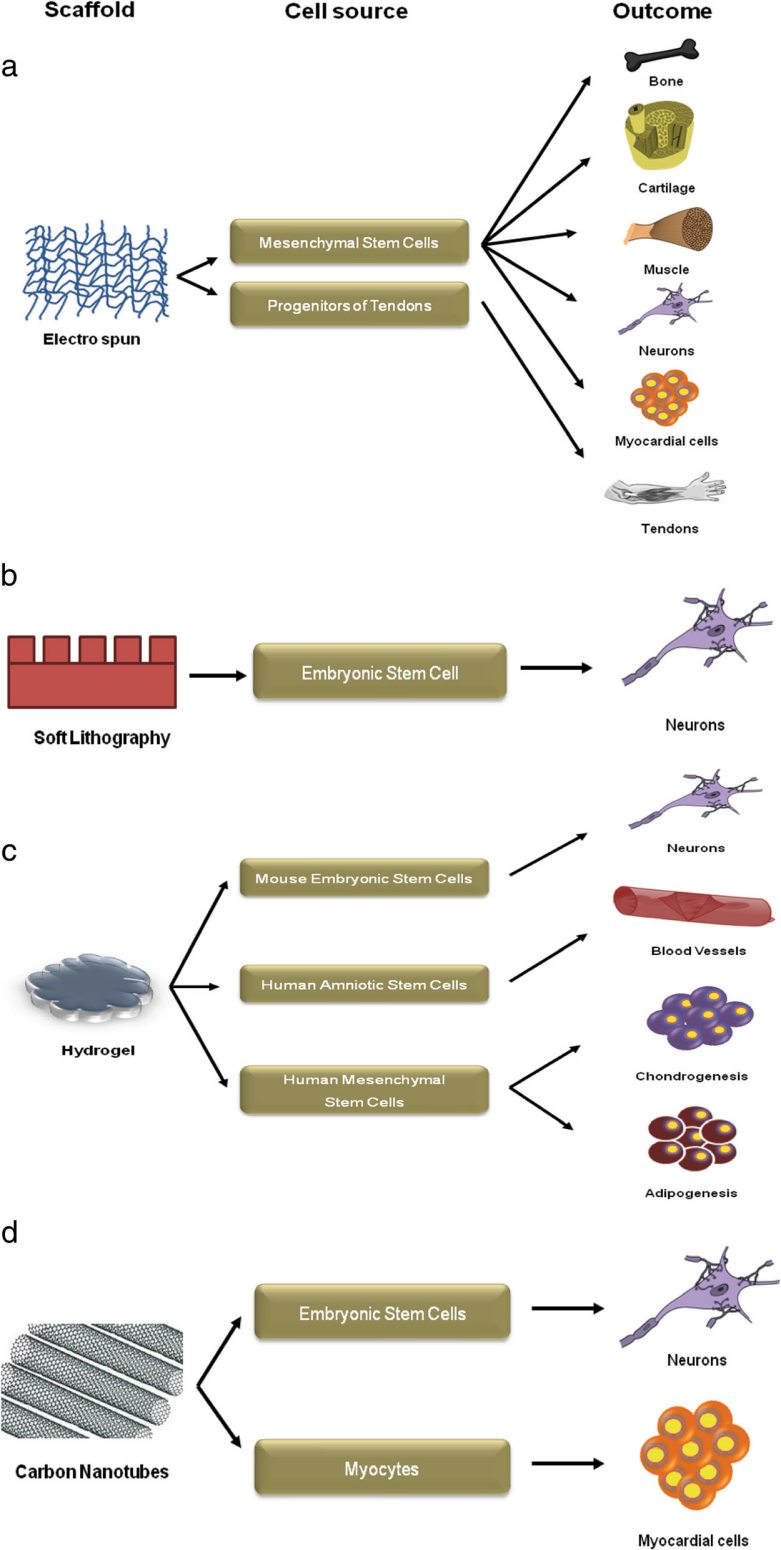
Various nanoscale platforms for directing stem cell fate. Scaffolds with (**a**) nanofibrous architecture, (**b**) soft lithography, (**c**) hydrogel, and (**d**) carbon nanotubes. These microenvironmental cues direct stem cell differentiation to a specific lineage

An amalgamation of methods with a microengineered platform comprising of a soft hydrogel can be used for inducing differentiation of stem cells (Fig. 3). The outcome of stem cell lineage specification has been tabulated with specific biomaterials in Table 1.

**Table 1:** Strategic mode of directing the fate of stem cells through nanotopography of the synthetic scaffolds

No.	Cells	Scaffolds	Differentiated cell type
1	Rat hair follicle stem cells (HFSCs)	Aligned poly-ε-caprolactone (PCL) nanofiber	Neuronal lineage
2	Mouse embryonic stem cells (ESCs)	Poly-l-lactic acid (PLLA) nanofiber	Osteogenic lineage
3	Retinal progenitor cells (RPCs)	Microfabricated PCL	Differentiated RPC
4	Mouse ESCs	Aligned PCL nanofibers	Neuronal lineage
5	Human mesenchymal stem cells (MSCs)	Type 1 collagen nanofiber	Osteogenic differentiation
6	Human MSCs	Poly(lactide-co-glycolide) nanofiber	Osteogenic lineage
7	Rat mesenchymal stem cells (MSCs)	PCL nanofiber	Osteogenesis
8	Human MSCs	Polydimethylsiloxane (PDMS) nanogroove	Neuronal lineage
9	Human MSCs	Polyacrylamide hydrogel	Neuronal lineage
10	Rat hippocampal progenitor cells	Micropatterned polystyrene with laminin	Neuronal lineage

## Biophysical regulators of stem cell fate

Stem cells respond to biophysical cues using cell signaling crosstalk, receptors and ligand interaction, protein modifications, protein-protein interactions, and transcriptional and translational regulations. The cell membrane enveloping the nanofeatures can result in increased intramembranous tension and rearrangement of cortical cytoskeleton thereby influencing cell morphology and behavior. Various mechanotransduction pathways have been proposed, such as the MAPK, the PI3K/Akt, RhoA/ROCK, Wnt/β-catenin, and the TGF-β pathways that rely mostly on the interaction of the cell with its biophysical environment. Significantly, all these mechanotransduction pathways are coupled with many other potent growth factor-mediated signaling pathways to regulate stem cell fate.

### Mechanotransduction pathways to regulate stem cell fate

As an external signal, nanotopographical features of the ECM is capable of governing stem cell fate determination, but how this biophysical cue is translated into intracellular signaling remains elusive. Stem cells sense and respond to these insoluble biophysical signals through integrin-mediated adhesions and the interplay between integrin molecules is a controlling step in signal transduction. The force balance between the endogenous cytoskeleton contractility and external mechanical forces that are transmitted across cell ECM adhesions regulate such signaling. Through the arrangement of adhesion epitopes available to the cell, the topographical features on the substrates such as grooves/pillars of micrometer to nanometer size are sensed by cells [89, 90]. The size of the adhesive area is the most significant physical signal to determine cell fate as it is mediated by the integrins [91]. The various integrin-mediated signaling mechanisms are described below.

#### MAPK pathway

The Ras/MAPK pathway is activated when the biophysical signal is through integrin-mediated focal adhesion signaling. The key molecules that participate in this mechanotransduction system are focal adhesion kinase and Src family kinases (fyn) [92–94]. Furthermore, the Ras-Raf-MEK-ERK pathway gets triggered, but the exact molecular mechanism is not well known yet. Several possible pathways have been postulated, such as integrin-FAK-Grb2-SOS-Ras [95], integrin-fyn-Shc-Grb2-SOS-Ras [96], through the epidermal growth factor (EGF) receptor [97]. The MAPK pathway plays a critical role during the different stages of stem cell differentiation; for instance, temporal MAPK signaling dictates adipocyte differentiation [98]. Neural stem cells spontaneously differentiate into neurons when cultured on hydrogen terminated ultra-nanocrystalline diamond films with fibronectin integrin beat-1, focal adhesion kinase, and the MAPK pathway plays a decisive role [99]. Osteoblast differentiation on micro/nanotextured topography and titanium implant surface as well as magnesium alloy coated with porous b-tricalcium phosphate is modulated by the activation of the MAPK pathway [100, 101]. Gold nanoparticles interact with the cell membrane of MSCs inducing mechanical stress, thereby activating p38 MAPK signaling which promotes osteogenic specific gene expression in lieu of adipogenic signals [102]. Hence, MAPK signaling pathway plays a vital role in spatial and temporal differentiation of stem cells.

#### PI3K/Akt

This is a downstream pathway of Ras, and can also be activated through the integrin-mediated signaling in both embryonic stem cells and somatic stem cells [103–105]. Pharmacological blockage of the PI3K pathway reduces the expression of Nanog, a key transcription factor of pluripotency [106]. Mechanical strain induces integrin activation mediated by the PI3K pathway, enabling the binding of integrins to ECM proteins to activate the further downstream functionality [107]. In response to the extracellular signals, PI3K is crucial for inducing critical alteration to determine the cellular functions. An enumerate transcription factor, kinase, and regulatory molecule activity becomes regulated upon the phosphorylation of the key downstream effector of PI3K, i.e., serine-threonine kinase [108]. Upon binding to the cell surface receptors, growth factors such as insulin-like growth factor (IGF)-1 and neurotrophins (NGF) can trigger the activation of the PI3K pathway promoting survival and self-renewal of stem cells [109]. Woo et al. generated a composite scaffold of hydroxyapatite and polymer PLLA to study the properties of MSCs which survived better in the presence of an increased P13/Akt activity [110]. Zhang et al. also showed that a scaffold of PCL attached with Arg-Gly-Asp enhanced the proliferation of MSCs with the activation of the PI3K/Akt pathway via integrin [111]. Schwann cells cultured on carboxymethylated chitosan reveal that the proliferation is regulated by the intracellular signaling mechanism of Erk1/2 and PI3/Akt kinase pathways [112]. Mesenchymal stem cells under mechanical strain induced bone morphogenetic protein (BMP)2 that could be blocked in the presence of blockers of PI3 kinase [113]. Composite scaffolds of porous β-calcium silicate with poly-d,l-lactide-glycolide enhance the osteogenic and angiogenic potential of MSCs and endothelial cells by recruitment of AMP-activated protein kinase, Erk1/2 and PI3K/Akt pathways [114]. Polylactide-co-glycolide scaffolds impregnated with fibronectin and type I collagen induce osteogenic lineage of cultured MSCs via MAPK and PI3 kinase pathways [115].

#### RhoA/ROCK

RhoA acts through Rho-kinase (ROCK) and is a key molecular regulator of actin cytoskeleton tension and focal adhesion formation. By the activation of focal adhesion kinase through integrin-mediated signaling, this pathway acts as a downstream target [116]. RhoA can be activated by different growth factors and cytokines as well as biophysical signals from the cellular microenvironment [117]. High RhoA activity associated with high actomyosin contractility induces osteogenesis, while low RhoA activity leads to adipogenesis [118]. Parekh et al. have demonstrated that the osteogenic differentiation of hBMSCs in 2D polyethylene glycol hydrogel in the absence of supplements is triggered through elevated expression levels of actin and myosin filaments [119]. The RhoA/ROCK pathway influences the stem cell differentiation through the regulation of Sox-9 as the transcription factor [120].

#### Wnt/β-catenin

Wnt/β-catenin signaling regulates the fate decisions of almost all stem cell types in a spatio-temporal regulated manner. For example, a dosage-dependent Wnt signaling results in either maintenance of the pluripotency or promotion of neural differentiation [121]. Biophysical signals have been shown to directly regulate Wnt signaling as demonstrated in osteoblasts in a time-dependent manner [122]. The signaling crosstalk between Wnt and integrin signaling has been postulated through two independent frameworks, where integrin-linked kinases and focal adhesion kinases are ascertained to play a significant role [123]. Both the pathways promote the accumulation and translocation of β-catenin in the nucleus independently [124].

## Conclusions

Tissue engineering is a promising field that has been developing intensely due to its potential for clinical application in cellular maintenance/differentiation. Scaffold functionalization tuned for specific application and cell response is the targeted approach. Despite advances in the development of biomimetic nanofibrous scaffolds for tissue engineering applications, several challenges still remain. Various factors from the extracellular environment known to control cell adhesion, proliferation, and differentiation have been incorporated into the design of biomaterials to achieve the objective of creating increased communication between biomaterials and their surrounding biological environment. The effects of these material modifications on cell activity are dose dependent as well as spatio-temporal dependent. One of the major challenges is to develop complex clinically relevant 3D porous scaffolds using a composite combination of materials. Additionally, it is crucial to develop a strategy to produce fibers with a diameter identical to that of native ECM fibers while maintaining high porosity for cell infiltration and migration. Research is now focused on developing an efficient biocompatible scaffold using combinatorial approaches, which could pave the way for developing scaffolds mimicking tissue junctions such as neuromuscular junctions and bone-cartilage junctions. Developing combinatorial signaling crosstalk with a dosage gradient would be a big asset in the branch of developmental biology where the axis, orientation, as well as polarization of the cells and matrix are important. These scaffolds could be used for studies related to gradient signaling, which is essential in tissue junctions. These areas of new and expanding research demonstrate the vastness as well as the challenges encountered in this multidisciplinary field. Newer technologies can offer better clinical outcomes and expand commercial arenas.

## Abbreviations

2D: Two-dimensional
3D: Three-dimensional
BMP: Bone morphogenetic protein
CNT: Carbon nanotube
ECM: Extracellular matrix
FGF: Fibroblast growth factor
hBMSC: Human bone marrow stromal cell
IGF: Insulin-like growth factor
MAPK: Mitogen-activated protein kinase
mESC: Mouse embryonic stem cell
MSC: Mesenchymal stem cell
MWNT: Multi-walled carbon nanotube
NGF: Neurotrophins
P13K/Akt: Phosphoinositide 3-kinase/protein kinase B
PCL: Poly-ε-caprolactone
PDMS: Polydimethylsiloxane
PGA: Polyglycolic acid
PLA: Polylactic acid
PLGA: Polylactic-co-glycolic acid
PLLA: Poly-l-lactic acid
RhoA/ROCK: Rho-associated protein kinase
SWNT: Single-walled carbon nanotube
TGF: Transforming growth factor
